# Comprehensive analysis of long non-coding RNAs highlights their spatio-temporal expression patterns and evolutional conservation in *Sus scrofa*

**DOI:** 10.1038/srep43166

**Published:** 2017-02-24

**Authors:** Zhonglin Tang, Yang Wu, Yalan Yang, Yu-Cheng T. Yang, Zishuai Wang, Jiapei Yuan, Yang Yang, Chaoju Hua, Xinhao Fan, Guanglin Niu, Yubo Zhang, Zhi John Lu, Kui Li

**Affiliations:** 1 State Key Laboratory of Animal Nutrition, Institute of Animal Science, Chinese Academy of Agricultural Sciences, Beijing 100193, China; 2 Agricultural Genome Institute at Shenzhen, Chinese Academy of Agricultural Sciences, Shenzhen, 518124, China; 3 MOE Key Laboratory of Bioinformatics, Center for Synthetic and Systems Biology, Center for Plant Biology and Tsinghua-Peking Joint Center for Life Sciences, School of Life Sciences, Tsinghua University, Beijing 100084, China

## Abstract

Despite modest sequence conservation and rapid evolution, long non-coding RNAs (lncRNAs) appear to be conserved in expression pattern and function. However, analysis of lncRNAs across tissues and developmental stages remains largely uncharacterized in mammals. Here, we systematically investigated the lncRNAs of the Guizhou miniature pig (*Sus scrofa*), which was widely used as biomedical model. We performed RNA sequencing across 9 organs and 3 developmental skeletal muscle, and developed a filtering pipeline to identify 10,813 lncRNAs (9,075 novel). Conservation patterns analysis revealed that 57% of pig lncRNAs showed homology to humans and mice based on genome alignment. 5,455 lncRNAs exhibited typical hallmarks of regulatory molecules, such as high spatio-temporal specificity. Notably, conserved lncRNAs exhibited higher tissue specificity than pig*-*specific lncRNAs and were significantly enriched in testis and ovary. Weighted co-expression network analysis revealed a set of conserved lncRNAs that are likely involved in postnatal muscle development. Based on the high degree of similarity in the structure, organization, and dynamic expression of pig lncRNAs compared with human and mouse lncRNAs, we propose that these lncRNAs play an important role in organ physiology and development in mammals. Our results provide a resource for studying animal evolution, morphological complexity, breeding, and biomedical research.

Intensive transcriptome sequencing, also known as deep sequencing, has led to the discovery that mammalian genomes encode a vast range of non–protein-coding RNAs (ncRNAs) that differ in size and level of conservation^1^,^2^. The proportion of ncRNAs in an organism’s genome has a direct correlation with its developmental complexity^3^. ncRNAs are generally classified into many different RNA types, including microRNA (miRNAs), Piwi-interacting RNAs (piRNAs), small nucleolar RNAs (snoRNAs), small interfering RNAs (siRNAs), and long noncoding RNA (lncRNAs). LncRNAs are defined as transcribed RNA fragments >200 bp, and they do not have open reading frames of >100 amino acids. Several recent studies have shown mammalian lncRNAs to be heterogeneous and diverse as well as critically important in cellular function, development, and disease via their transcriptional and posttranscriptional regulation of gene expression^4^. In this study, we aimed to further elucidate the origin, evolution, and function of mammalian ncRNAs, the genome of *S. scrofa*^5^, a species widely used in medical research, by analyzing the content and function of its lncRNA.

LncRNAs have long been ascribed an important role in the evolution of complex traits, and recent studies have revealed that thousands of lncRNAs are evolutionarily conserved in mammals, though not to the same extent as many protein-coding genes^6^,^7^. Compared with protein-coding genes, however, lncRNAs exhibit lower expression levels and more precise tissue-specific (i.e., spatial) or developmental stage-specific (i.e., temporal) expression patterns^7^. Because of their highly restricted expression patterns, further identification and functional analysis of lncRNAs among diverse species, tissues, and cell types is needed. Despite genome-wide identification of lncRNAs in *Homo sapiens, Mus musculus, Danio rerio, Caenorhabditis elegans, Oncorhynchus mykiss (rainbow trout),* and *Arabidopsis thaliana*^8^,^9^,^10^,^11^,^12^,^13^, comprehensive expression profiling across organs and developmental stages has not been conducted for mammals.

The domestic pig (*Sus scrofa*) has a close and complex relationship with humans for at least 10,000 years^5^. Compared to traditional rodent models, the miniature pig model is more similar to humans in body size, growth, development, immunity, physiology, and metabolism, as well as genome sequence^5^,^14^. The miniature pig model is widely used in biomedical research including cardiology, pharmacology, oncology, aging, and other areas of studies^15^. However, compared to the mouse model as well as humans, knowledge regarding the pig transcriptome across organs and developmental stages is very limited. In fact, little is known about the spatial and temporal expression, evolution, and function of lncRNAs in the miniature pig.

In this study, we sought to comprehensively analyze miniature pig lncRNAs across multiple organs and developmental stages. We first sequenced two mixed libraries based on rRNA depleted total RNA sequencing from 110 different tissue samples from 3 pig breeds. Then we performed strand-specific rRNA depletion sequencing across 9 organs and at 3 postnatal developmental stages of skeletal muscle in Guizhou miniature pigs. We predicted 10,813 lncRNAs with high confidence. Notably, the lncRNA of *Sus scrofa* are remarkably similar to that of their mammalian counterparts. Conserved lncRNAs showed higher tissue specificity than *S. scrofa*-specific lncRNAs and were enriched in the testis and ovary. We also identified a set of conserved lncRNAs that are likely involved in skeletal muscle development. Overall, we obtained the first comprehensive expression profile of lncRNA across multiple organs and developmental stages in *S. scrofa*. These data support further annotation of the pig genome and will facilitate the use of the miniature pig model in biomedical research.

## Result

### Genome-wide and conservative cataloging of *S. scrofa* lncRNAs

**Figure 1:**
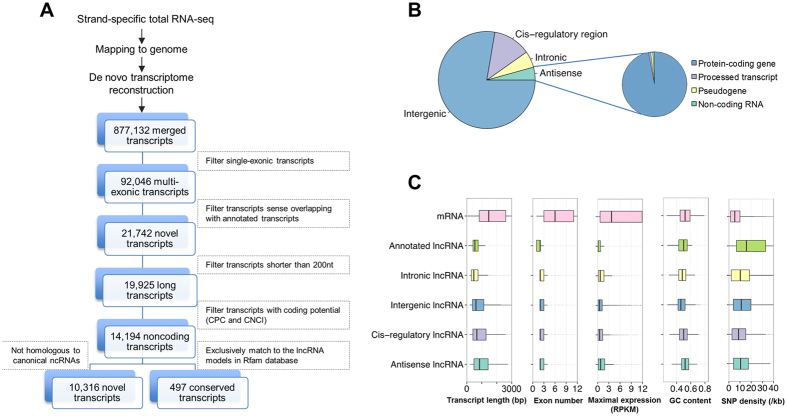
Identification, classification, and characterization of novel lncRNAs in *S. scrofa*. (**A**) Pipeline for the identification of novel lncRNAs. (**B**) Statistics of lncRNAs in different categories according to their genomic locations. (**C**) Comparison of transcript length, exon number, maximum expression level, GC content, and SNP density between lncRNAs and mRNAs.

To systematically identify lncRNAs and profile their spatio-temporal expressions in *S. scrofa*, we conducted pair-end and strand-specific sequencing to confirm gene orientation and to predict antisense transcripts (see Methods). We constructed a total of 13 RNA libraries for 2 pooled samples (derived from 110 tissues samples from 3 breeds, 14 organs, and 27 developmental stages; Supplementary Table S1), 9 organs (adipose, heart, kidney, liver, lung, ovary, spleen, testis, and skeletal muscle at 240 days after birth) and two additional skeletal muscle at 0 and 30 days after birth. We obtained >1.2 billon sequencing reads; to our knowledge, this is the deepest RNA sequencing of *S. scrofa* to date (Supplementary Table S2). To comprehensively profile the lncRNAs, we reconstructed the consensus transcriptome of *S. scrofa* based on the large pool of sequencing data, using TopHat mapping^16^ followed by Cufflinks assembly^17^ (see Methods). This procedure resulted in a comprehensive *de novo* assembly of 877,132 transcripts (Fig. 1a), and model transcripts were well assembled (Supplementary Table S3).

To identify the lncRNAs, we developed a highly stringent pipeline that used 6 hierarchical filtering steps (Fig. 1a). This filter removed annotated, short, and unreliable transcripts, as well as those having the potential to encode proteins, leaving 14,194 noncoding transcripts. Using Rfam^18^ and other structural RNAs databases^19^,^20^,^21^, we correlated these transcripts according to their sequential or structural homology with canonical structural RNAs (e.g., rRNA, tRNA, snRNA, snoRNA, etc.). We preserved transcripts that exclusively matched conserved lncRNA model. With this comprehensive yet conservative pipeline, we identified a final set of 10,813 *S. scrofa* lncRNAs (including 9,075 novel lncRNAs) located at 8,266 loci (Fig. 1a) (Supplementary Table S4). We also calculated the average fold change per lncRNA in each sample, and found that the median of the maximum value across 13 samples was 13.1 fold (Supplementary Fig. S1). Besides, 18% of lncRNAs were located in the genomic region with CpG island annotation (Supplementary Table S4).

### *S. scrofa* lncRNAs share similarities with their mammalian counterparts

Stranded RNA sequencing can directly assign the orientations of novel transcripts; thus, the newly identified lncRNAs were classified into four categories (Supplementary Fig. S2). First, the majority of identified lncRNAs (8,398; 78%) were identified as lincRNAs located far away (at least 2 kb) from annotated transcripts (intergenic, Fig. 1b). The remaining three categories were defined as follows. We found that 463 *S. scrofa* lncRNAs (4%) were identified as antisense transcripts that overlapped the exonic regions of annotated genes (mostly protein-coding genes) in the opposite strand (antisense, Fig. 1b), 591 *S. scrofa* lncRNAs (5%) were located in the intronic regions of annotated genes (intronic, Fig. 1b), and the remaining 1,361 *S. scrofa* lncRNAs (13%) were located within 2 kb upstream or downstream of annotated genes and were designated as lncRNAs in *cis*-regulatory regions (*cis*-regulatory region, Fig. 1b). This categorization is consistent with previous studies of the humans and other mammalian genomes^8^,^22^.

To determine the similarities of *S. scrofa* lncRNAs with their mammalian counterparts, we analyzed the primary characteristics of these lncRNAs (Fig. 1c). The average length of *S. scrofa* lncRNAs (1,051 nt) was significantly shorter than that of protein-coding genes, namely mRNA, which has an average length of 1,983 nt. In addition, *S. scrofa* lncRNAs contained fewer exons (average, 2.5) than mRNAs (average, 8.7). We then compared the expression abundance of the 3 types of transcripts defined above. These properties still hold when comparing mRNAs and lncRNAs in a fixed expression level (Supplementary Fig. S3). The maximum reads per kilobase per million mapped reads (RPKM) values of the 11 tissue samples were considered to represent their respective expression levels. As shown in the box plots, *S. scrofa* lncRNAs (average RPKM_max_ = 2.5) were much less abundant than mRNAs (average RPKM_max_ = 21.3). The newly identified lncRNAs and annotated lncRNAs were expressed at levels comparable to those of other mammals^9^,^23^. Our sequencing data were based on total RNA libraries that did not include a selection step for poly(A)^+^ RNAs; thus, the relative differences in the abundances of the 3 types of transcripts reflected by these datasets were different from those based on poly(A)^+^ -enriched RNA sequence data. In agreement with previous studies in plants and animals, we found that lncRNAs are generally shorter, have fewer exons, and have lower expression levels than protein-coding genes^8^,^24^,^25^.

Guanine-cytosine (GC) content is important for the strand stability of DNA/RNA, whereas single-nucleotide polymorphisms (SNPs) reveal sequence variation, evolutionary conservation, and natural selection. We compared GC content and SNP density among different types of transcripts. The average GC content of novel lncRNA transcripts (47.8%) was significantly lower than that of mRNAs (52.2%) and was similar to the set of 47 annotated lncRNAs (48.3%). And it was higher than that of random regions (41.6%), intergenic (41.7%) and the whole-genome (42.3%). Meanwhile, as expected, the GC content of intergenic lncRNAs (47.2%) was lower than that of antisense (51.8%), *cis*-regulatory (49.9%), and intronic (47.8%) lncRNAs. We identified 148,699 SNPs in 9,147 lncRNAs and 372,730 SNPs in 21,452 mRNA transcripts based on the *S. scrofa* dbSNP database (build 140)^26^. The density of SNPs in novel lncRNAs (15.47/kb) was significantly higher than in protein-coding genes (8.57/kb), whereas the SNP density of the 47 previously annotated *S. scrofa* lncRNAs was even higher (22.84/kb) because they are mostly located at intergenic regions (Fig. 1c).

### *S. scrofa* lncRNAs show spatio-temporally restricted expression patterns

**Figure 2:**
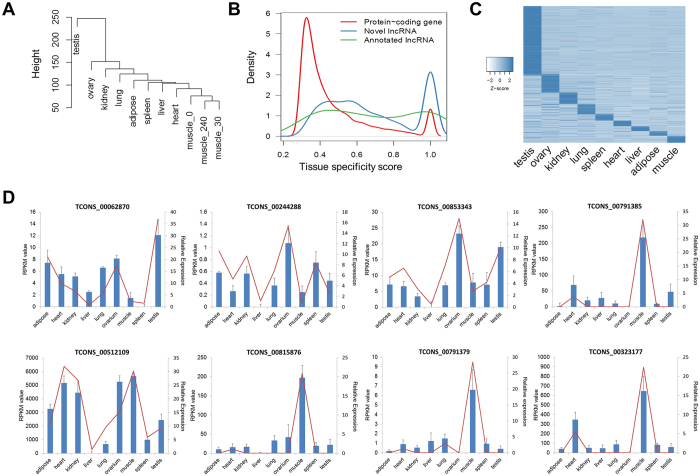
Tissue specificity of *S. scrofa* lncRNAs. (**A**) A tree of expression correlations between samples. Correlations were calculated using the expression levels of lncRNAs in 11 tissue samples (9 organs and 3 developmental stages of skeletal muscle). (**B**) The tissue-specificity scores of novel lncRNAs compared with those of annotated lncRNAs and protein-coding genes. (**C**) Heat map of the expression of tissue-specific lncRNAs. **(D)** qPCR validation of the expression levels in 9 tissues for 8 randomly selected *S. scrofa* lncRNAs (blue bar and Y-axis on the right). Error bars indicate standard deviation (SD) based on three biological replicates. The RPKM values of the lncRNAs from RNA-seq data were also shown (red line and Y-axis on the left).

LncRNAs tend to be expressed in both tissue-specific (i.e., spatial) and stage-specific (i.e., temporal) manners in animals^9^,^11^,^27^. To confirm that this is the case for *S. scrofa* lncRNAs, we analyzed RNA sequence data from 11 tissues for temporally and spatially restricted lncRNA expression. First, we conducted a cluster analysis of 11 tissue samples, and the results of this analysis revealed significant clustering with biological relevance (Fig. 2a). And the clustering result is statistically robust (Supplementary Fig. S4). The lncRNA expression in skeletal muscle samples collected at three postnatal developmental stages (day 0, day 30, and day 240) demonstrated clustering. Different tissue samples with similar cell components also showed clustering. For example, heart samples clustered with skeletal muscle samples. This result was as expected because heart tissue mainly consists of cardiac muscle, which is quite similar to skeletal muscle. Interestingly, however, reproductive tissues, specifically testis and ovary, clustered separately from other somatic tissues. The result indicates that distinct populations of lncRNAs may be involved in somatic and germ-line processes.

Next, we evaluated tissue-specific patterns of *S. scrofa* lncRNAs based on Jensen-Shannon divergence as previously described^11^. Agreement with the data from other species (human body map) (Supplementary Fig. S5), the density plot of tissue specificity score exhibited two peaks: genes around the left peak had low tissue-specificity scores; genes around the right peak had high tissue-specificity scores, which mean that they were only expressed in a few samples. Compared with protein-coding genes, the newly identified lncRNAs demonstrated significantly higher tissue specificity (Fig. 2b). Overall, 56.1% of *S. scrofa* lncRNAs were expressed (RPKM >0) in <3 samples, while only 4.8% of *S. scrofa* lncRNAs were constitutively expressed in all 11 tissue samples. In contrast, only 19.2% of protein-coding genes were detected in <3 samples, while 48.4% of protein-coding genes were ubiquitously expressed. We also showed that lncRNAs still had higher tissue specificity than protein-coding genes in a fixed expression level (Supplementary Fig. S6). The expression patterns of lncRNAs potentially related to the tissue-specific regulatory roles of these transcripts, similar to those of lncRNAs in other mammals.

We analyzed each lncRNA enriching a specific tissue to determine its relative expression pattern^28^. In total, we detected 5,455 tissue-specific lncRNAs (50.4%) (Supplementary Table S5), and most of these (4,998; 91.6%) were highly restricted to a single tissue type. Testis and ovary tissue contained the most tissue-specific lncRNAs (68.6%), as these samples exhibited a distinct structure due to enrichment of germ cells (Fig. 2c). The lncRNAs in the testis tissue comprised the largest cluster on the heat map plot; this result is concordant with the dominant expression of lncRNAs in the testes of other mammals^29^,^30^. This results are also consistent with a previous study in salmonid, where the highest number of lncRNAs predicted in *rainbow trout* is also specifically expressed in testis^13^. It showed that the expression pattern across organs maybe highly conserved. The lncRNAs in the ovary tissue comprised the second largest cluster of lncRNAs expression. These results highlight the potential role of lncRNAs in the reproduction system. Taken together, these analyses indicate that lncRNAs have spatially restricted expression patterns.

To confirm the spatial expression patterns of *S. scrofa* lncRNAs, we randomly selected 18 newly identified lncRNAs, 10 with RT-PCR and 8 with qPCR, to validate their expression levels in nine tissues. We found good concordance between the RT-PCR and qPCR results and the RNA sequencing data (Fig. 2d, Supplementary Fig. S7), suggesting that the lncRNA expression patterns based on RNA sequencing analysis are reliable.

### *S. scrofa* lncRNAs exhibit high sequence conservation with *H. sapiens* and *M. musculus*

**Figure 3:**
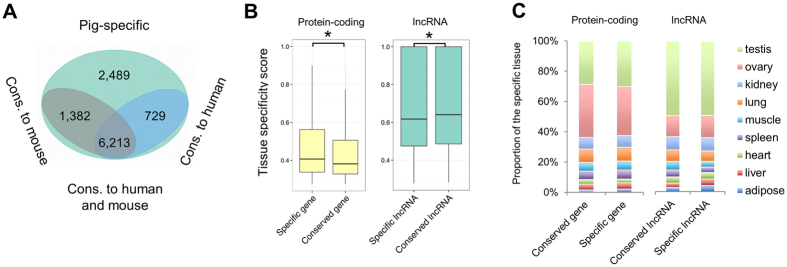
Evolutionary conservation of lncRNAs in *S. scrofa*. (**A**) Classes of lncRNAs with different levels of conservation in *H. sapiens* and *M. musculus*. (**B**) Comparison of the tissue specificity of conserved and specific lncRNAs using protein-coding genes as controls. (**C**) Comparison of the tissue specificity of *S. scrofa* lncRNAs and protein-coding genes.

Although lncRNAs have generally low levels of sequence conservation and exhibit rapid evolution in vertebrates, several studies have supported an evolutionarily conserved role of lncRNA in mammals^27^,^29^. Because mouse and pig models are widely used for human biomedical research, we further assessed evolutionarily conservation of *S. scrofa* lncRNAs in the mouse and human genomes by performing pairwise alignments between *S. scrofa* and other species to identify conserved lncRNAs. As shown in Fig. 3a, we classified the lncRNAs of *S. scrofa* into four groups^30^. Overall, 729 lncRNAs (6.7%) were conserved only in *S. scrofa* and *H. sapiens*; 1,382 lncRNAs (12.8%) were conserved only between *S. scrofa* and *M. musculus*; 6,213 lncRNAs (57.5%) were conserved in *S. scrofa, H. sapiens*, and *M. musculus*; and 2,489 *S. scrofa*-specific lncRNAs (23.0%) were not conserved in *H. sapiens* or *M. musculus* (Supplementary Table S4). We adopted the method used by a previous study^30^ to define conserved lncRNA and our results were comparable to theirs. In addition, protein-coding transcripts and intronic DNAs were similarly compared. The conservation level of *S. scrofa* lncRNAs was comparable to that of intronic sequences, but it was substantially lower than that of protein-coding transcripts (Supplementary Fig. S8a,b). Besides, we also detected transcript-level homology of our predicted lncRNAs with active transcribed lncRNAs in human and mouse^31^, and found that 45% and 29% of our lncRNAs can be aligned to human and mouse lncRNAs, respectively, which is also quite analogous to a previous study^32^ (Supplementary Table S4). Furthermore, more than 60% of lncRNAs with transcript-level homology were also considered as conserved in genome alignment analysis (Supplementary Fig. S8c,d).

The tissue specificity of lncRNAs has been highly correlated with their evolutionary dynamics^7^,^30^. Therefore, we compared the tissue-specificity scores of *S. scrofa*–specific lncRNAs and lncRNAs conserved in *S. scrofa, H. sapiens*, and *M. musculus* using protein-coding genes as controls. The lncRNAs conserved across species tended to have higher tissue specificity than the *S. scrofa*–specific lncRNAs, and this was not the case for protein-coding genes (Fig. 3b). Interestingly, testis tissue contained the highest proportion of conserved lncRNAs (~50%) (Fig. 3c). It was supposed that conserved, tissue-specific lncRNAs significantly may contribute to maintaining testis physiology and to postnatal development. Other recent studies have revealed that the testis is a rich source of many unique lncRNA transcripts^33^,^34^ and that the testis has the fastest rate of evolution in lncRNAs in tetrapods^7^.

### *S. scrofa* lncRNAs are dynamically expressed during postnatal skeletal muscle development

**Figure 4:**
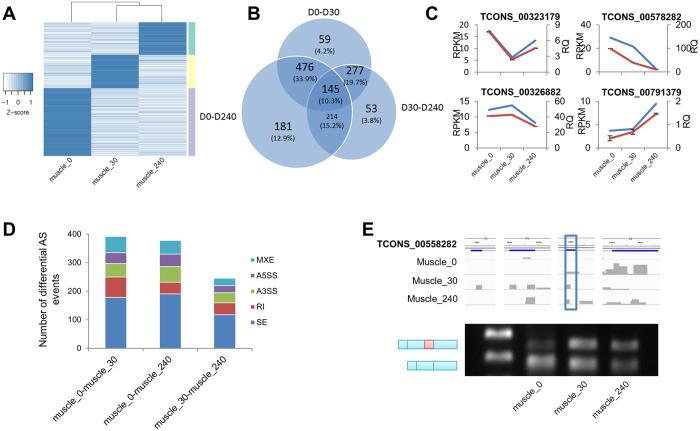
Differential expression and splicing of *S. scrofa* lncRNAs during skeletal muscle development. (**A**) Differential expression of *S. scrofa* lncRNAs among 3 developmental stages of skeletal muscle based on RNA sequencing data (Q value < 0.05). Muscle_0,_30 and_240 means skeletal muscle at day 0, day 30, and day 240 after birth. Each row represents data for one lncRNA. LncRNAs were clustered using hierarchical clustering. Gray indicates high expression level; light indicates low expression (see Supplementary Table S6 for the IDs of these differentially expressed lncRNAs). (**B**) Venn diagram representing the number and proportion of differentially expressed lncRNAs detected at each the developmental stage; D0, D30, and D240 indicate day 0, day 30 and day 240 after birth. (**C**) Validation of differential lncRNA expression by qPCR (red line with error bars). The RPKM value from deep sequencing data is shown for comparison (blue line). (**D**) Statistics of differential splicing events (*S. scrofa* lncRNAs and protein-coding genes) during muscle development based on RNA sequencing data, including mutually exclusive exons (MXEs), alternative 5′ splice sites (A5SSs), alternative 3′ splice sites (A3SSs), retained introns (RIs), and skipped exons (SEs). (**E**) Validation of differential splicing of a *S. scrofa* lncRNA by RT-PCR.

Many recent reports have concluded that lncRNAs markedly contribute to the development of skeletal muscle^35^,^36^. To investigate whether dynamically expressed lncRNAs were associated skeletal muscle development and to discover which lncRNAs potentially regulate skeletal muscle development, we profiled the temporal expression of *S. scrofa* lncRNAs in postnatal skeletal muscle at 3 different developmental stages (day 0, day 30 days, and day 240 after birth). This time series allowed us to follow the expression dynamics of lncRNAs and protein-coding genes as development proceeded^9^,^37^. In this analysis, we detected 1,405 lncRNAs that exhibited significant changes in expression level between any two of the three developmental stages we evaluated (q-value < 0.05) (see a full list in Supplementary Table S6). Among these, 714 lncRNAs (>50%) were specifically and highly expressed in skeletal muscle at day 0 after birth. Skeletal muscle samples at day 30 and day 240 showed similar lncRNA transcription profiles; 689 differentially expressed lncRNAs were detected between these 2 developmental stages (Fig. 4a). In contrast, a significant change in expression profile was evident between skeletal muscles tissue samples at day 0 and day 30; 957 lncRNAs were differentially expressed between these 2 developmental stages (Fig. 4a). A Venn diagram representing the number and proportion of differentially expressed lncRNAs detected at each of the developmental stage transitions was shown in Fig. 4b. Meanwhile, we used Gfold^38^, a statistic tool designed for detecting differentially expressed genes when no biological replicates were available. We found that nearly a half of differentially expressed genes defined by DEGseq were also considered as significant at a cutoff of Gfold value 1 (Supplementary Table S6). Together, these results suggest that *S. scrofa* lncRNAs are dynamically expressed in a temporal manner and are involved in postnatal skeletal muscle development. Meanwhile, we randomly selected 10 differentially expressed lncRNAs and performed quantitative polymerase chain reaction (qPCR) to validate the expression patterns based on the samples used in the RNA sequencing analysis (Fig. 4c, Supplementary Fig. S9). The qPCR results were quite concordant with those of the RNA sequencing. Thus, our study has provided a cross-identified set of *S. scrofa* lncRNAs that might function in skeletal muscle development.

LncRNAs are known to be co-expressed and functional related with their overlapped and/or neighboring protein coding genes^7^,^9^. So we calculated the expressional correlation between the differentially expressed intergenic lncRNAs and their neighboring protein-coding genes, and observed a positive correlate expression pattern (mean Pearson correlation coefficient, r = 0.386) between the lncRNA-neighbor gene pairs. We also observed that most of the differentially expressed (DE) intragenic lncRNAs (67%) had a positive correlation with their overlapped protein coding genes, but the correlation coefficient (r = 0.229) was lower that of intergenic lncRNAs, which might be caused by only a few intragenic lncRNAs (n = 165) were differentially expressed in skeletal muscle. This trend is similar with previous studies in human, mouse, and rainbow trout^11^,^12^,^22^. We analyzed the Gene Ontology (GO) term enrichment and Kyoto Encyclopedia of Genes and Genomes (KEGG) pathway of these protein-coding genes. The neighbors of lncRNAs differentially expressed in skeletal muscle tissue between day 0 and day 30 were significantly enriched with 31 GO terms known to be involved in anterior/posterior pattern formation, acetyl-CoA catabolic process, tricarboxylic acid cycle, coenzyme catabolic processes, and other functions (Supplementary Table S7; P < 0.05). The results of our KEGG pathway analysis suggested that these significantly enriched protein-coding neighbors were involved in lysine degradation (P = 0.019), citrate cycle (TCA cycle) (P = 0.062), and pyruvate metabolism (P = 0.096). Protein-coding neighbors of lncRNAs differentially expressed in skeletal muscle between day 30 and day 240 were significantly associated with 12 GO terms involved in the regulation of developmental growth, muscle contraction, muscle system processes, and ER-associated protein catabolic processes (Supplementary Table S8; P < 0.05). Unfortunately, the protein-coding neighbors were not significantly enriched in any KEGG pathway.

### *S. scrofa* lncRNAs rarely demonstrate differentially alternative splicing compared with protein-coding genes in postnatal skeletal muscle

Previous studies have suggested that >90% of multi-exonic protein-coding genes can be alternatively spliced, giving rise to distinct isoforms across different temporal stages^39^. Using our deep RNA sequencing data regarding *S. scrofa* lncRNAs and protein-coding genes in postnatal skeletal muscle, we identified 5 types of major alternative splicing events: mutually exclusive exons (MXEs), alternative 5′ splice sites (A5SSs), alternative 3′ splice sites (A3SSs), retained introns, and skipped exons. As alternatively splicing events are frequently stochastic, we only reported statistically significant events. Hundreds of differential alternative splicing events were detected across the 3 temporal stages of skeletal muscle development we analyzed (Fig. 4d). Among these, skipped exons were the most frequent. For protein-coding gene, it may cause frame-shifting thus altered the gene function. For lncRNA without open reading frame, there are currently limited experimental reports. By theory, it could remove specific proteins’ binding sites, or change the global RNA structure.

Consistent with our differential expression analysis, most of these events occurred in the early developmental stage. That is, more differential alternative splicing events were detected between day 0 and day 30 and between day 0 and day 240 than were found between day 30 and day 240.

Consistent with alternative splicing analyses in other mammals^40^, differential alternative splicing events mostly occurred among protein-coding genes (Supplementary Fig. S10a) that were significantly enriched in GO terms related to muscle development, including myofibrils, contractile fibers, sarcomeres, and others (see Supplementary Fig. S10b). And the differential alternative splicing events occurred quite rarely among lncRNAs (3 skipped exons, 3 A5SS, 1 A3SS, 1 retained intron, and 1 MXE) (Supplementary Table S9), perhaps because they contained fewer exons, or because their functioning is mainly on the genome instead of cytoplasm. Finally, we validated an lncRNA candidate with a skipped exon in its third exon at the early developmental stage (day 0), TCONS_00558282, using RT-PCR (Fig. 4e) and Sanger sequencing (Supplementary Fig. S11). These results confirm our identification of differential alternative splicing events.

### Co-expression network of conserved lncRNAs and protein-coding genes

Co-expression of lncRNA and protein-coding genes can indicate functional relatedness or regulatory relationships^7^,^9^. Because co-expression may also arise spuriously, we focused only on conserved lncRNAs and protein-coding genes in order to remove false positives^7^,^10^. Because our network analysis required a large number of samples, we added 6 newly sequenced *S. scrofa* ovary samples and 13 published *S. scrofa* datasets (Supplementary Table S10). All Additional samples and our initial 11 tissue samples (except the 2 pooled samples) were processed identically (see Methods) to yield a large expression matrix of 5,003 lncRNAs and 9,653 protein-coding genes in 30 RNA sequence samples.

**Figure 5:**
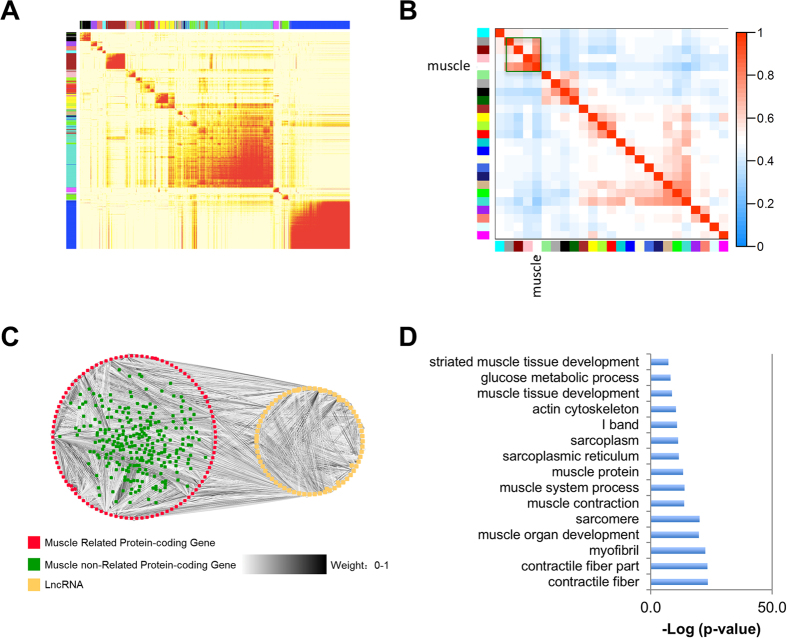
Weighted co-expression network of conserved *S. scrofa* lncRNAs and protein-coding genes. (**A**) The global weighted co-expression network of conserved pig lncRNAs and protein-coding genes shown as a heat-map plot of the topological matrix. In the plot, each row and column corresponds to a gene; the sides are colored according to the module to which it belongs. In the heat map, a light color denotes low topological overlap, i.e., weak co-expression, whereas darker colors denote high topological overlap, i.e., stronger co-expression. Dark squares along the diagonal correspond to the modules. (**B**) Correlation plot of 25 module eigengenes and the muscle vector. Each row and column in the heat map corresponds to 1 module eigengene (labeled by the same color with (**A**) or the muscle vector (specifically indicated by the word). In the heat map, red color represents high adjacency (positive correlation) and blue color represents low adjacency (negative correlation). The squares of red color along the diagonal represent the meta-modules (modules with similar expression patterns). The largest module related to skeletal muscle development (pink module) is shown in green rectangle. (**C**) Co-expressed lncRNAs and protein-coding genes in the largest module that are closely related to skeletal muscle development. (**D**) Enriched GO terms for the protein-coding genes in the module shown in (**C**).

To determine the likely function of lncRNAs in skeletal muscle development, we performed a weighted gene co-expression network analysis (WGCNA)^41^ (see Material and Methods). The whole network was constructed based on a topological matrix and has been clustering into 25 interconnected gene modules (Fig. 5a, Supplementary Fig. S12). We found that at least 3 modules, especially the pink module, is highly correlated to the muscle development according to the correlation plot (Fig. 5b) (see Material and Methods). The representative lncRNAs and genes that show co-expression with the eigengene in each module, as well as the enriched GO terms were shown in Supplementary Table S11. Furthermore, we also used the guilt-by-association’ strategy^23^ to predict the potential function for each lncRNA (see Material and Methods). The top 10 enriched GO terms and the correlated protein-coding genes for lncRNA were listed in Supplementary Table S12.

Next, we visualized the largest module related to skeletal muscle development (pink module), which consisted of 78 lncRNAs and 361 protein-coding genes (Fig. 5c, Supplementary Table S13). Among the 78 lncRNAs, 64 showed significant correlation with muscles and 23 of them were hubs. Although closely associated at expression level, co-expressed genes/lncRNAs were not closely located in the genome. For instance, 12.8% of lncRNAs in the largest module related to muscle development had no protein-coding genes located in the same chromosome. For the remaining 87.2%, the average distance between lncRNAs and their closest protein-coding genes is 3.6 MB. The co-expression network was able to predict functional relatedness, as illustrated by the high frequency of connections within gene ontology (GO) categories^10^. Our GO enrichment analysis revealed that *S. scrofa* lncRNAs that were co-expressed with protein-coding genes mainly involved in skeletal muscle development processes, such as contractile fiber, muscle organ development, muscle contraction, and glucose metabolic process (Fig. 5d). Therefore, the 78 *S. scrofa* lncRNAs in the module were potentially important for postnatal skeletal muscle development because their expression patterns were strongly correlated with those of known muscle-related genes. Notably, the weighted co-expression network is unsigned; in other words, these 78 lncRNAs may be expressed in a coordinate or reverse manner with genes related to muscle development. These results suggested putative regulatory functions for a subset of *S. scrofa* lncRNAs in postnatal skeletal muscle development.

## Discussion

LncRNAs are involved in various biological processes via diverse mechanisms^42^. However, because of their tissue-type and cell-type specificity, the definition of lncRNAs is evolving as the discovery of lncRNAs continues. The miniature pig, *S. scofa*, a widely used biomedical model for *H. sapiens*, has attracted increasing attention in recent years. In the present study, we performed strand-specific total RNA sequencing on a series of representative tissues, and we systematically identified 10,813 *S. scrofa* lncRNAs in the miniature pig. We confirmed a large portion of lncRNAs predicted by previous studies (Supplementary Fig. S13)^32^,^43^ and we identified 9,075 novel lncRNAs in *S. scrofa*. Moreover, we classified our predicted lncRNAs into categories. And we should point out that most (65.5%) of the lncRNAs in the *cis*-regulatory regions were antisense to the transcription start site of annotated genes. And sense transcripts maybe 5′ or 3′ exon of known mRNAs as a result of the limited completeness of pig genome annotation.

In contrast with previous studies, our sequencing samples have provided strand information; our sequencing samples included 11 tissue samples and 2 comprehensive libraries generated from 110 samples from several *S. scrofa* breeds for different tissue types at several developmental stages (Supplementary Table S1). In addition, our total RNA libraries facilitated the identification of both poly(A)^+^ lncRNAs and non-poly(A)^+^ lncRNAs. Based on these samples, we systematically identified and characterized *S. scrofa* lncRNAs. However, we could not tell which one has polyA tail or not from our data. Studies on polyadenylation of lncRNA were currently an emerging field with a large number of questions awaited to be answer. LncRNAs predicted from this study will contribute positively to further studies.

The genomic characteristics of *S. scrofa* lncRNAs, including short length and low expression level, are quite similar to those of lncRNAs in other mammals^9^,^27^. A notable feature of lncRNAs is their strong tissue specificity, and our repository of *S. scrofa* lncRNAs successfully recapitulated their divergence among tissue samples. However, we should point out that further validation on the tissue specificity of individual lncRNA should be perform as our data is lacking of biological replicates. Moreover, 57% of the newly identified lncRNAs in *H. sapiens* and *M. musculus* based on genome alignment. These conserved lncRNAs showed higher tissue specificity than did *S. scrofa–*specific lncRNAs, suggesting that lncRNAs with strong tissue specificity may be more generally related to biological processes in their associated tissues. We also found that the reproductive organs, especially the testis, harbored the largest number of tissue-specific lncRNAs, which may indicate that *S. scrofa* lncRNAs play functionally important, although largely unknown, roles in spermatogenesis and ovogenesis. This finding is in accord with observations reported in lncRNA studies of other mammals^29^,^30^.

The most challenging obstacle in the analysis of lncRNAs is the determination of their biological functions. Many studies have demonstrated that lncRNAs play critical roles in tissue physiology and organogenesis in tissue-specific and stage-specific manners. We focused on lncRNAs associated with skeletal muscle development. Skeletal muscle is an important motor organ in animals and humans. It undergoes dramatic changes with aging, including hypertrophy, loss of muscle mass, reduced strength, and impaired regenerative ability. Therefore, identification of lncRNAs associated with skeletal muscle development and the determination of their specific biological functions will support animal breeding as well as biomedical research on muscle-related diseases and aging. Previous studies have shown that lncRNAs play an important role in myogenesis; specifically, linRNA-MD1^44^ and H19^45^ have also been associated with skeletal muscle development.

In the present study, we first detected 1,405 differentially expressed lncRNAs during postnatal skeletal muscle development in the miniature pig. Most were expressed during the early developmental stage, which implies that most lncRNAs play roles in early muscle development. We also performed differential splicing analysis of *S. scrofa* lncRNAs and protein-coding genes. For protein-coding genes, alternative splicing is a regulated process during gene expression that results in a single gene encoding multiple proteins. Differential splicing events in protein-coding genes were observed most often in the early developmental stage. Among lncRNAs, however, differential splicing events were quite rare. Our study provides a rich resource of lncRNAs that will facilitate future studies of skeletal muscle development in mammals and muscle-related disease.

Despite modest sequence conservation and rapid evolution in animal species in general, lncRNAs appear to conserved both spatially and temporally in expression and function. To better understand the regulatory role of lncRNAs in skeletal muscle development, we performed a weighted co-expression network analysis. Although the number of samples included in our study was sufficient for lncRNA annotation and the aforementioned analysis, it was inadequate for network analysis. Therefore, we added extra tissue samples from other pig breeds (Supplementary Table S10), and we identified modules from the weighted co-expression network that correlated most strongly with muscle samples. We selected a large module that was highly related to muscle development through correlation analysis (Fig. 5c) and found that the protein-coding genes in the module were significantly enriched in genes related to muscle development, including contractile fibers, myofibrils, sarcomeres, etc. The co-expression patterns of the lncRNAs in the same module also indicated that these lncRNAs are likely functionally related to muscle development. It is worth noting that co-expression associations are indirect evidences that need lots of efforts on experimental validations. To ascertain the biological role of individual lncRNA, more functional studies are needed.

## Conclusion

We generated a comprehensive *S. scrofa* lncRNAs BodyMap for the Guizhou miniature pigs, which have undergone a high degree of artificial selection and are widely used as an animal model in biomedical research. Our study revealed a large number of lncRNAs with spatial and temporal expression patterns. The roles of lncRNAs in mammalian evolution and human muscle-related disease are not yet fully understood. Our analysis provides a valuable resource for future studies in mammalian evolution and biomedical research in which the miniature pigs are used as a large animal model.

## Material and Methods

### Collection of tissue samples

All pigs were raised under the same environment at our farm and were sacrificed at a commercial slaughter house. Tissue samples were collected at postnatal day 240. *Longissimus dorsi* samples were collected at postnatal days 0, 30, and 240. In addition, we prepared 2 mixed RNA libraries derived from more than 110 samples from various breeds, different tissues types, and several developmental stages. Samples from 3 individuals were harvested as biological replicates. Tissue samples were manually dissected from each animal and were rapidly frozen in liquid nitrogen. All animal procedures were performed according to protocols approved by the Biological Studies Animal Care and Use Committee in Beijing Province, China.

### Total RNA sequencing of *S. scrofa* tissue samples

Total RNA was isolated using TRIzol Reagent (Invitrogen, Carlsbad, CA, USA). Genomic DNA was removed using DNaseI (Qiagen, Beijing, China). The quantity and quality of the RNA were assessed using an Agilent 2100 Bioanalyzer (Agilent Technologies, CA, USA). Ribosomal RNA was depleted using a Ribo-Zero Magnetic Kit (Epicentre, Madison, WI, USA). Mixed libraries were constructed by mixing equal quantities of each RNA sample. Strand-specific libraries for paired-end sequencing were prepared using SMART or dUTP protocols. Libraries were sequenced on the Illumina Genome Analyzer II (2 mixed samples) or the HiSeq 2500 platform (11 tissue samples).

### *S. scrofa* transcriptome reconstruction

Adaptors in the total RNA sequencing reads were subjected to quality trimming using custom scripts. Processed reads from each sample were aligned with the reference genome of *S. scrofa* (v10.2) using TopHat (v1.3.2)^16^. Parameters were set for strand-specific mapping (i.e., library-type ‘fr-second strand’ for the SMART protocol and ‘fr-firststrand’ for the dUTP protocol). To facilitate the alignments, annotations from Ensembl were provided for each TopHat run. Mapped reads from each sample were assembled into transcripts independently using Cufflinks (v1.3.0)^17^ with the assistance of known annotations. Putative transcripts were retrieved using the parameter ‘–min-frags-per-transfrag 3’^11^. Finally, assembled transcripts from each sample were merged into a consensus transcriptome using Cuffmerge^17^.

### Novel *S. scrofa* lncRNA identification

The consensus transcriptome in *S. scrofa* was further subjected to a series of stringent filtering steps. First, we retained only multi-exonic transcripts for further analysis to avoid unreliable transcripts owing to the complexity of transcriptional reconstruction. This strategy was commonly used in many previous studies^9^,^11^. Next, we filtered transcripts overlapping with annotated elements and short transcripts with lengths <200 nt. Subsequently, we removed all transcripts with coding potential using the Coding–Non-Coding Index (CNCI)^46^ and the Coding Potential Calculator (CPC)^47^ with default parameters. Finally, we eliminated all transcripts homologous to canonical ncRNAs stored in the following databases: miRBase^19^, tRNAdb^20^, snoRNAbase^21^, and Rfam^18^ using BLAST^48^ (i.e., a sequence similarity search) and Infernal (a structure similarity search)^49^. Currently, 225 Rfam models are available for conserved long non-coding RNAs; therefore, we retrieved transcripts that exclusively matched those models. Finally, the remaining transcripts were annotated as lncRNAs.

### Tissue-specificity analysis

Tissue-specificity scores were calculated based on the Jensen-Shannon divergence between the actual expression levels of transcripts across 11 tissue samples and a predefined extreme expression pattern (only expressed in 1 sample)^11^. For each transcript, the associated tissues were defined according to the expression of the most highly restricted lncRNAs according to both the absolute RPKM values and the relative expression levels measured as *Z* scores. For the cutoffs, we used *Z* score ≥1.5 and RPKM ≥0.5, which corresponded to a 3-fold coverage according to our sequencing depth for defining associated tissues^11^. Transcripts specifically expressed in either developmental stage of skeletal muscle were considered to be associated with skeletal muscle.

### Conservation analysis

We used two methods to define conserved lncRNAs. The first method is based on genome alignment. To map *S. scrofa* transcripts to other genomes, we used pairwise alignments produced by the UCSC comparative genomics pipeline^30^,^50^. Then, we analyzed the files in overlapping chain format and defined a sequence-level conserved lncRNA when 50% of its nucleotides uniquely intersected with an alignment in the chain file (coverage > = 50%). Other lncRNAs were denoted as *S. scrofa*-specific lncRNAs if they did not overlap any alignments in the chain file.

In addition, we defined the conserved lncRNAs with another method based on Blast results of transcripts. We aligned the newly identified lncRNAs with active transcribed lncRNAs in human and mouse^31^ by Blast using parameters ‘-task blastn -word_size 6 -evalue 0.01 -strand plus’, which were adapted from previous studies^27^. We also required that the length of BLAST hits should be exceed 20% of query sequences (i.e. coverage > = 20%). These were called transcript-level conserved lncRNAs.

### Differential expression/splicing analysis

We used htseq-count^51^ to count the reads in *S. scrofa* lncRNAs and protein-coding genes; this procedure required strand-specific counting (-s yes for SMART and -s reverse for dUTP) and ≥1 mapping quality. We then calculated the RPKM (reads per kilobases per million mapped reads; counted on read pairs in case of paired ends) values accordingly. We used DEGseq (MARS method)^52^ for differential expression analysis of these results. *S. scofa* lncRNAs and protein-coding genes showing a fold change ≥2 and *q* < 0.05 were considered to be differentially expressed. The *q* values were adjusted using the BH method. Meanwhile, we used Gfold to rank the differentially expressed genes^38^.

We conducted rigorous TopHat mapping twice using the splice-site information from each sample. Alternative splicing events were identified using MATS and a FDR (false discovery rate) cutoff of 5% was required^53^. Differential splicing analysis was performed in a pairwise manner among muscle samples.

### Weighted co-expression network

We used 30 RNA sequence samples for network construction. And conserved lncRNAs and genes were selected based on the genome alignment. After TopHat mapping, the reads in each lncRNA/protein-coding gene were calculated using htseq-count, and RPKM values were then calculated accordingly. Based on the expression matrix, we constructed a weighted co-expression network using the R package WGCNA^41^. First, an adjacency matrix was constructed based on the calculation of pairwise Pearson correlation coefficients; a power value of 6 was chosen as the soft threshold to maximize the fitness to the scale-free topology of the whole network. Next, we calculated the topological overlap matrix based on the adjacency matrix, and we clustered the genes into distinct modules using hierarchical clustering followed by dynamic tree cutting. This analysis yielded 25 modules containing genes with coordinated expression patterns.

For each module, we defined the first principal component as the gene expression profile for each gene in the module; these components were designated the eigengenes according to WGCNA terminology. To determine the module most relevant for skeletal muscle development, we defined a vector to encode the muscle tissue samples (encoded as 1) and other tissue samples (encoded as 0). We referred to this vector as the muscle vector. We then correlated the eigengenes of each module with the muscle vector, and higher correlations indicated that the module was related to muscle development. Because the GO annotation for the genes in *S. scrofa* is relatively limited, we converted the gene IDs into their human homologs based on the TreeFam database^54^, and we performed GO enrichment analysis based on human annotation using the DAVID web server^55^.

Furthermore, we used the ‘guilt-by-association’ strategy^23^ to infer the putative function of each lncRNA based on the co-expression network. Firstly, we retrieved the protein-coding genes significantly correlated with each lncRNA, and then we used these protein-coding genes (required the number is no less than 30) to conduct GO enrichment analysis.

### RT-PCR and RT-qPCR

Total RNA for RT-PCR and quantitative real-time PCR (RT-qPCR) was extracted as described for RNA sequencing. First-strand cDNA fragments were obtained by reverse transcription using the ImPro-IITM Reverse Transcription System (Promega, Madison, WI, USA). RT-PCR was performed using routine PCR programs (Tm = 60 °C) with 35 amplification cycles. The RT-qPCR reaction was performed on a 7500 FAST Real-Time PCR System (Applied Biosystems, Foster City, CA, USA) according to the SYBR Premix Ex Taq^TM^ instructions. All reactions were replicated three times. Expression levels of transcripts encoding glyceraldehyde 3-phosphate dehydrogenase (*GAPDH*), β-actin (*ACTB*), and hypoxanthine-guanine phosphoribosyltransferase (*HPRT*) were detected as endogenous control measurements. The expression levels of all genes of interest were normalized to those of the control genes using the 2^−ΔΔ^Ct method. All primer information is listed in Supplementary Table S14.

### Data deposition

The RNA sequencing data were deposited in the Gene Expression Omnibus database under the accession codes GSE73763 and GSE73593.

## Additional Information

**How to cite this article:** Tang, Z. *et al*. Comprehensive analysis of long non-coding RNAs highlights their spatio-temporal expression patterns and evolutional conservation in *Sus scrofa. Sci. Rep.* **7**, 43166; doi: 10.1038/srep43166 (2017).

**Publisher's note:** Springer Nature remains neutral with regard to jurisdictional claims in published maps and institutional affiliations.

## Supplementary Material

Supplementary Figures

Supplementary Tables

## References

[b1] Eddy S. R. Non-coding RNA genes and the modern RNA world. Nat Rev Genet 2, 919–29 (2001). 11733745 10.1038/35103511

[b2] Dinger M. E. . Long noncoding RNAs in mouse embryonic stem cell pluripotency and differentiation. Genome Res 18, 1433–45 (2008). 18562676 10.1101/gr.078378.108 PMC2527704

[b3] Taft R. J., Pheasant M. & Mattick J. S. The relationship between non‐protein‐coding DNA and eukaryotic complexity. Bioessays 29, 288–299 (2007). 17295292 10.1002/bies.20544

[b4] Wang S. . Long noncoding RNA H19 inhibits the proliferation of fetal liver cells and the Wnt signaling pathway. FEBS Lett (2016). 10.1002/1873-3468.12078 26801864

[b5] Groenen M. A. . Analyses of pig genomes provide insight into porcine demography and evolution. Nature 491, 393–398 (2012). 23151582 10.1038/nature11622 PMC3566564

[b6] Kutter C. . Rapid turnover of long noncoding RNAs and the evolution of gene expression. PLoS Genet 8, e1002841 (2012). 22844254 10.1371/journal.pgen.1002841 PMC3406015

[b7] Necsulea A. . The evolution of lncRNA repertoires and expression patterns in tetrapods. Nature 505, 635–40 (2014). 24463510 10.1038/nature12943

[b8] Derrien T. . The GENCODE v7 catalog of human long noncoding RNAs: analysis of their gene structure, evolution, and expression. Genome research 22, 1775–89 (2012). 22955988 10.1101/gr.132159.111 PMC3431493

[b9] Pauli A. . Systematic identification of long noncoding RNAs expressed during zebrafish embryogenesis. Genome research 22, 577–591 (2012). 22110045 10.1101/gr.133009.111 PMC3290793

[b10] Natoli G. & Andrau J. C. Noncoding transcription at enhancers: general principles and functional models. Annu Rev Genet 46, 1–19 (2012). 22905871 10.1146/annurev-genet-110711-155459

[b11] Cabili M. N. . Integrative annotation of human large intergenic noncoding RNAs reveals global properties and specific subclasses. Genes & development 25, 1915–1927 (2011). 21890647 10.1101/gad.17446611 PMC3185964

[b12] Paneru B., Al-Tobasei R., Palti Y., Wiens G. D. & Salem M. Differential expression of long non-coding RNAs in three genetic lines of rainbow trout in response to infection with Flavobacterium psychrophilum. Scientific Reports 6, 36032 (2016). 27786264 10.1038/srep36032 PMC5081542

[b13] Al-Tobasei R., Paneru B. & Salem M. Genome-Wide Discovery of Long Non-Coding RNAs in Rainbow Trout. Plos One 11 (2016). 10.1371/journal.pone.0148940 PMC4764514 26895175

[b14] Wall R. & Shani M. Are animal models as good as we think? Theriogenology 69, 2–9 (2008). 17988725 10.1016/j.theriogenology.2007.09.030

[b15] Lunney J. K. Advances in swine biomedical model genomics. Int J Biol Sci 3, 179–184 (2007). 17384736 10.7150/ijbs.3.179 PMC1802015

[b16] Trapnell C., Pachter L. & Salzberg S. L. TopHat: discovering splice junctions with RNA-Seq. Bioinformatics 25, 1105–11 (2009). 19289445 10.1093/bioinformatics/btp120 PMC2672628

[b17] Trapnell C. . Transcript assembly and quantification by RNA-Seq reveals unannotated transcripts and isoform switching during cell differentiation. Nat Biotechnol 28, 511–5 (2010). 20436464 10.1038/nbt.1621 PMC3146043

[b18] Daub J., Eberhardt R. Y., Tate J. G. & Burge S. W. Rfam: annotating families of non-coding RNA sequences. Methods Mol Biol 1269, 349–63 (2015). 25577390 10.1007/978-1-4939-2291-8_22

[b19] Kozomara A. & Griffiths-Jones S. miRBase: annotating high confidence microRNAs using deep sequencing data. Nucleic Acids Res 42, D68–73 (2014). 24275495 10.1093/nar/gkt1181 PMC3965103

[b20] Juhling F. . tRNAdb 2009: compilation of tRNA sequences and tRNA genes. Nucleic Acids Res 37, D159–62 (2009). 18957446 10.1093/nar/gkn772 PMC2686557

[b21] Lestrade L. & Weber M. J. snoRNA-LBME-db, a comprehensive database of human H/ACA and C/D box snoRNAs. Nucleic Acids Res 34, D158–62 (2006). 16381836 10.1093/nar/gkj002 PMC1347365

[b22] Zhang K., Huang K., Luo Y. & Li S. Identification and functional analysis of long non-coding RNAs in mouse cleavage stage embryonic development based on single cell transcriptome data. BMC genomics 15, 845 (2014). 25277336 10.1186/1471-2164-15-845 PMC4200203

[b23] Rinn J. L. & Chang H. Y. Genome regulation by long noncoding RNAs. Annu Rev Biochem 81, 145–166 (2012). 22663078 10.1146/annurev-biochem-051410-092902 PMC3858397

[b24] Guttman M. . Ab initio reconstruction of cell type-specific transcriptomes in mouse reveals the conserved multi-exonic structure of lincRNAs. Nat Biotechnol 28, 503–10 (2010). 20436462 10.1038/nbt.1633 PMC2868100

[b25] Li L. . Genome-wide discovery and characterization of maize long non-coding RNAs. Genome Biol 15, R40 (2014). 24576388 10.1186/gb-2014-15-2-r40 PMC4053991

[b26] Sherry S. T. . dbSNP: the NCBI database of genetic variation. Nucleic Acids Res 29, 308–11 (2001). 11125122 10.1093/nar/29.1.308 PMC29783

[b27] Ulitsky I., Shkumatava A., Jan C. H., Sive H. & Bartel D. P. Conserved function of lincRNAs in vertebrate embryonic development despite rapid sequence evolution. Cell 147, 1537–1550 (2011). 22196729 10.1016/j.cell.2011.11.055 PMC3376356

[b28] Li J. J., Huang H., Bickel P. J. & Brenner S. E. Comparison of D. melanogaster and C. elegans developmental stages, tissues, and cells by modENCODE RNA-seq data. Genome Res 24, 1086–101 (2014). 24985912 10.1101/gr.170100.113 PMC4079965

[b29] Hezroni H. . Principles of long noncoding RNA evolution derived from direct comparison of transcriptomes in 17 species. Cell Rep 11, 1110–22 (2015). 25959816 10.1016/j.celrep.2015.04.023 PMC4576741

[b30] Washietl S., Kellis M. & Garber M. Evolutionary dynamics and tissue specificity of human long noncoding RNAs in six mammals. Genome research 24, 616–28 (2014). 24429298 10.1101/gr.165035.113 PMC3975061

[b31] Zhao Y. . NONCODE 2016: an informative and valuable data source of long non-coding RNAs. Nucleic Acids Research 44, D203–D208 (2016). 26586799 10.1093/nar/gkv1252 PMC4702886

[b32] Zhou Z. Y. . Genome-wide identification of long intergenic noncoding RNA genes and their potential association with domestication in pigs. Genome biology and evolution 6, 1387–92 (2014). 24891613 10.1093/gbe/evu113 PMC4079208

[b33] Soumillon M. . Cellular source and mechanisms of high transcriptome complexity in the mammalian testis. Cell reports 3, 2179–2190 (2013). 23791531 10.1016/j.celrep.2013.05.031

[b34] Ward M., McEwan C., Mills J. D. & Janitz M. Conservation and tissue-specific transcription patterns of long noncoding RNAs. Journal of Human Transcriptome 1, 2–9 (2015). 27335896 10.3109/23324015.2015.1077591 PMC4894084

[b35] Nie M., Deng Z.-L., Liu J. & Wang D.-Z. Noncoding RNAs, Emerging Regulators of Skeletal Muscle Development and Diseases. BioMed research international 2015 (2015). 10.1155/2015/676575 PMC4516831 26258142

[b36] Gong C. . A Long Non-coding RNA, LncMyoD, Regulates Skeletal Muscle Differentiation by Blocking IMP2-Mediated mRNA Translation. Developmental cell 34, 181–191 (2015). 26143994 10.1016/j.devcel.2015.05.009

[b37] Gaiti F. . Dynamic and Widespread lncRNA Expression in a Sponge and the Origin of Animal Complexity. Mol Biol Evol 32, 2367–82 (2015). 25976353 10.1093/molbev/msv117 PMC4540969

[b38] Lipka A. E. . GAPIT: genome association and prediction integrated tool. Bioinformatics 28, 2397–2399 (2012). 22796960 10.1093/bioinformatics/bts444

[b39] Wang E. T. . Alternative isoform regulation in human tissue transcriptomes. Nature 456, 470–6 (2008). 18978772 10.1038/nature07509 PMC2593745

[b40] Tilgner H. . Deep sequencing of subcellular RNA fractions shows splicing to be predominantly co-transcriptional in the human genome but inefficient for lncRNAs. Genome Res 22, 1616–25 (2012). 22955974 10.1101/gr.134445.111 PMC3431479

[b41] Langfelder P. & Horvath S. WGCNA: an R package for weighted correlation network analysis. BMC Bioinformatics 9, 559 (2008). 19114008 10.1186/1471-2105-9-559 PMC2631488

[b42] Moran V. A., Perera R. J. & Khalil A. M. Emerging functional and mechanistic paradigms of mammalian long non-coding RNAs. Nucleic Acids Res 40, 6391–400 (2012). 22492512 10.1093/nar/gks296 PMC3413108

[b43] Zhao W. . Systematic identification and characterization of long intergenic non-coding RNAs in fetal porcine skeletal muscle development. Scientific Reports 5, 8957 (2015). 25753296 10.1038/srep08957 PMC4354164

[b44] Legnini I., Morlando M., Mangiavacchi A., Fatica A. & Bozzoni I. A feedforward regulatory loop between HuR and the long noncoding RNA linc-MD1 controls early phases of myogenesis. Mol Cell 53, 506–14 (2014). 24440503 10.1016/j.molcel.2013.12.012 PMC3919156

[b45] Dey B. K., Pfeifer K. & Dutta A. The H19 long noncoding RNA gives rise to microRNAs miR-675-3p and miR-675-5p to promote skeletal muscle differentiation and regeneration. Genes Dev 28, 491–501 (2014). 24532688 10.1101/gad.234419.113 PMC3950346

[b46] Sun L. . Utilizing sequence intrinsic composition to classify protein-coding and long non-coding transcripts. Nucleic Acids Res 41, e166 (2013). 23892401 10.1093/nar/gkt646 PMC3783192

[b47] Kong L. . CPC: assess the protein-coding potential of transcripts using sequence features and support vector machine. Nucleic Acids Res 35, W345–9 (2007). 17631615 10.1093/nar/gkm391 PMC1933232

[b48] Altschul S. F., Gish W., Miller W., Myers E. W. & Lipman D. J. Basic local alignment search tool. J Mol Biol 215, 403–10 (1990). 2231712 10.1016/S0022-2836(05)80360-2

[b49] Nawrocki E. P. & Eddy S. R. Infernal 1.1: 100-fold faster RNA homology searches. Bioinformatics 29, 2933–5 (2013). 24008419 10.1093/bioinformatics/btt509 PMC3810854

[b50] Rosenbloom K. R. . The UCSC Genome Browser database: 2015 update. Nucleic Acids Res 43, D670–81 (2015). 25428374 10.1093/nar/gku1177 PMC4383971

[b51] Anders S., Pyl P. T. & Huber W. HTSeq–a Python framework to work with high-throughput sequencing data. Bioinformatics 31, 166–9 (2015). 25260700 10.1093/bioinformatics/btu638 PMC4287950

[b52] Wang L., Feng Z., Wang X., Wang X. & Zhang X. DEGseq: an R package for identifying differentially expressed genes from RNA-seq data. Bioinformatics 26, 136–8 (2010). 19855105 10.1093/bioinformatics/btp612

[b53] Shen S. . MATS: a Bayesian framework for flexible detection of differential alternative splicing from RNA-Seq data. Nucleic Acids Res 40, e61 (2012). 22266656 10.1093/nar/gkr1291 PMC3333886

[b54] Li H. . TreeFam: a curated database of phylogenetic trees of animal gene families. Nucleic Acids Res 34, D572–80 (2006). 16381935 10.1093/nar/gkj118 PMC1347480

[b55] Huang D. W. . DAVID Bioinformatics Resources: expanded annotation database and novel algorithms to better extract biology from large gene lists. Nucleic Acids Res 35, W169–75 (2007). 17576678 10.1093/nar/gkm415 PMC1933169

