# Supplementary material for: Comprehensive analysis of long non-coding RNAs highlights their spatio-temporal expression patterns and evolutional conservation in Sus scrofa
Source: Sci Rep. 2017 Feb 24;7:43166. doi: 10.1038/srep43166 (PMC5324117; doi:10.1038/srep43166)
Supplement: Supplementary Figures [file srep43166-s1.doc]

**Supplementary Information**

**Comprehensive analysis of long non-coding RNAs highlights their spatio-temporal expression patterns and evolutional conservation in *Sus scrofa***

Zhonglin Tang^1,3‡*^, Yang Wu^2, ‡^, Yalan Yang^1,3, ‡^, Yu-Cheng T. Yang^2^, Zishuai Wang^1^, Jiapei Yuan^2^, Yang Yang^2^, Chaoju Hua^1^, Xinhao Fan^1^, Guanglin Niu^1^, Yubo Zhang^3^, Zhi John Lu^2*^ and Kui Li^1^,^3*^

^1^State Key Laboratory of Animal Nutrition, Institute of Animal Science, Chinese Academy of Agricultural Sciences, Beijing 100193, China.

^2^MOE Key Laboratory of Bioinformatics, Center for Synthetic and Systems Biology, Center for Plant Biology and Tsinghua-Peking Joint Center for Life Sciences, School of Life Sciences, Tsinghua University, Beijing 100084, China.

^3^Agricultural Genome Institute at Shenzhen, Chinese Academy of Agricultural Sciences, Shenzhen, 518124，China.

^‡^ These authors contributed equally to this work.

^*^To whom correspondence should be addressed:

Tel. +86 10-6283-3312. Fax. +86 10-6283-3312

E-mail: [tangzhonglin@caas.cn](mailto:tangzhonglin@caas.cn) (ZLT); [zhilu@tsinghua.edu.cn](mailto:zhilu@tsinghua.edu.cn) (ZJL); [likui@caas.cn](mailto:likui@caas.cn) (KL)

**Supplementary Figures**

# Supplementary Figure S1. Fold coverage along lncRNAs.


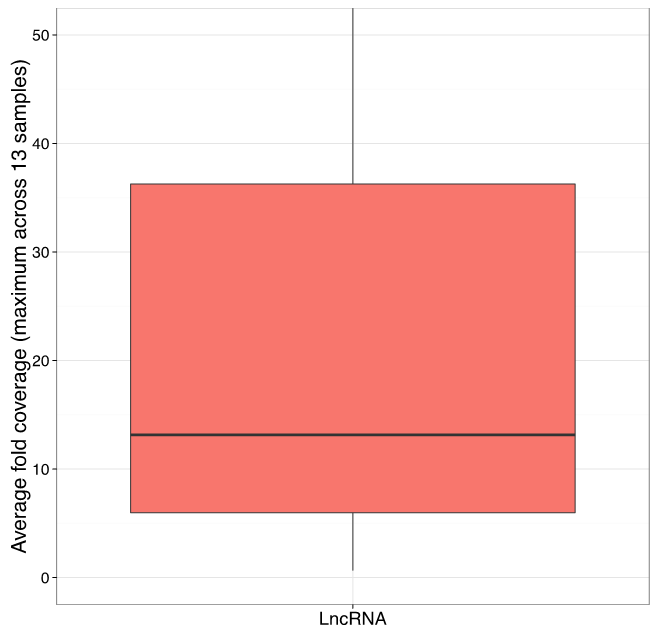


**Fold coverage along lncRNAs.** The maximum of the average fold coverage across 13 samples were calculated along newly predicted lncRNAs.

# Supplementary Figure S2. Scheme of classification of the lncRNAs in *Sus scrofa.*

**Scheme of classification of the lncRNAs in *Sus scrofa.*** LncRNAs are classified according to their relative relationships with surrounding genes. Antisense lncRNAs overlapped the exonic regions of their surrounding genes in the opposite strand. Intronic lncRNAs were located in the intronic regions of their surrounding genes, either in the same or opposite strand, and having no exonic overlap. LncRNAs in *cis*-regulatory regions are defined as the lncRNAs overlapping with the 2k upstream or downstream regions of their surrounding genes, either in the same or opposite strand, but having no exonic overlap. All other transcripts are classified as intergenic lncRNAs, which are far away from their surrounding genes.

# Supplementary Figure S3. Comparison of novel lncRNAs and mRNAs at a fixed expression level.


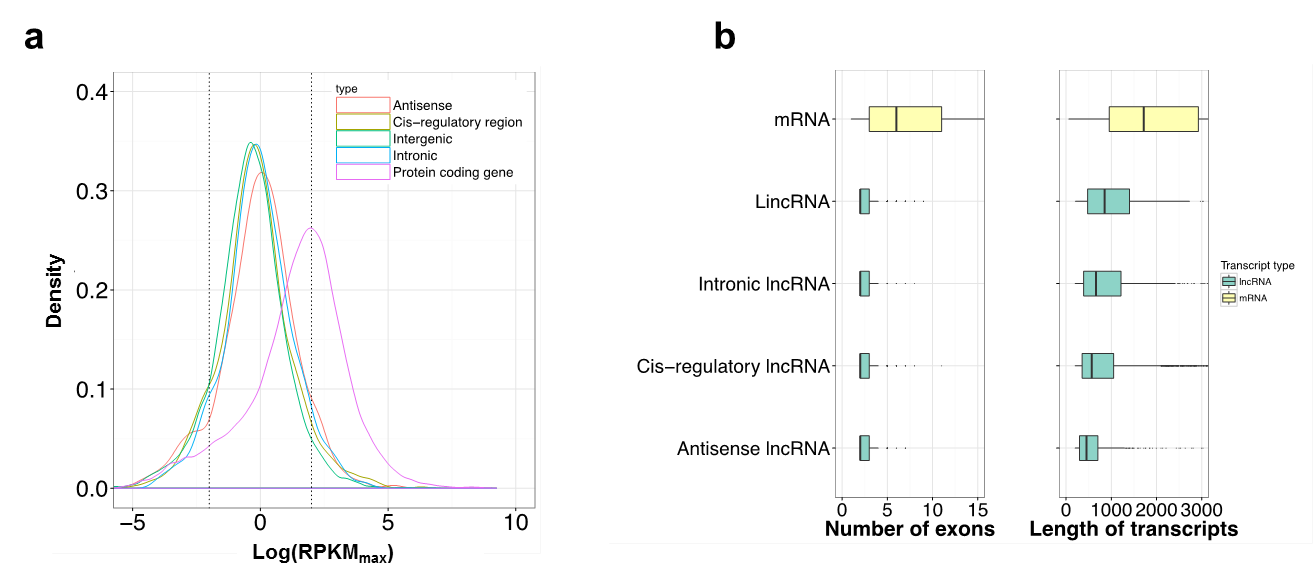


**Comparison of novel lncRNAs and mRNAs at a fixed expression level. (a)** The distribution of the maximum expression level, log(RPKM_max_), for lncRNAs and mRNAs was shown. And a fixed expression level (-2 < log(RPKM_max_) < 2) were displayed as vertical lines. **(b)** Comparison of exon number and transcript length between lncRNAs and mRNAs at a fixed expression level.

# Supplementary Figure S4. Clustering of tissue samples based on lncRNA expression.


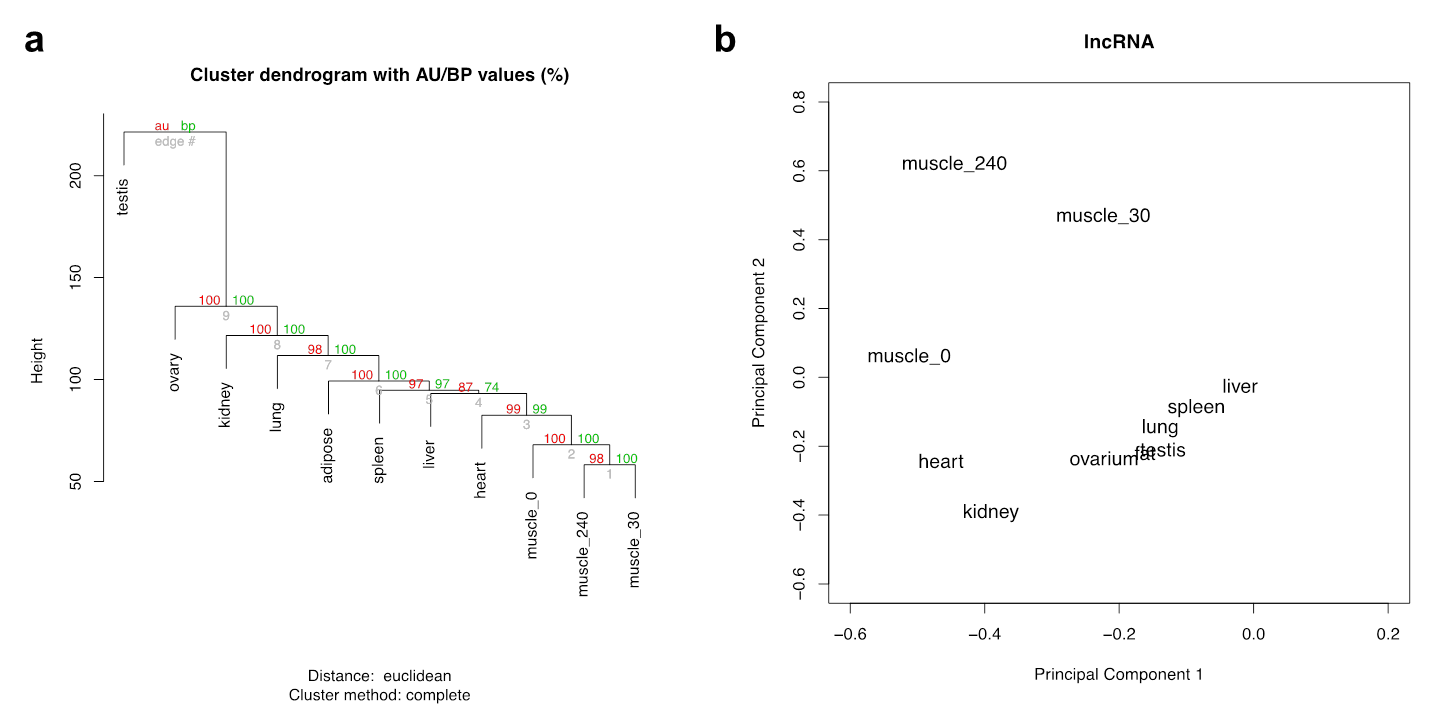


**Clustering of tissue samples based on lncRNA expression. (a)** A clustering tree with bootstrap p-values (AU p-values, red, left, BP p-values, green, right). **(b)** The PCA plot based on lncRNA expression.

# Supplementary Figure S5. Tissue specificity of human protein-coding genes.


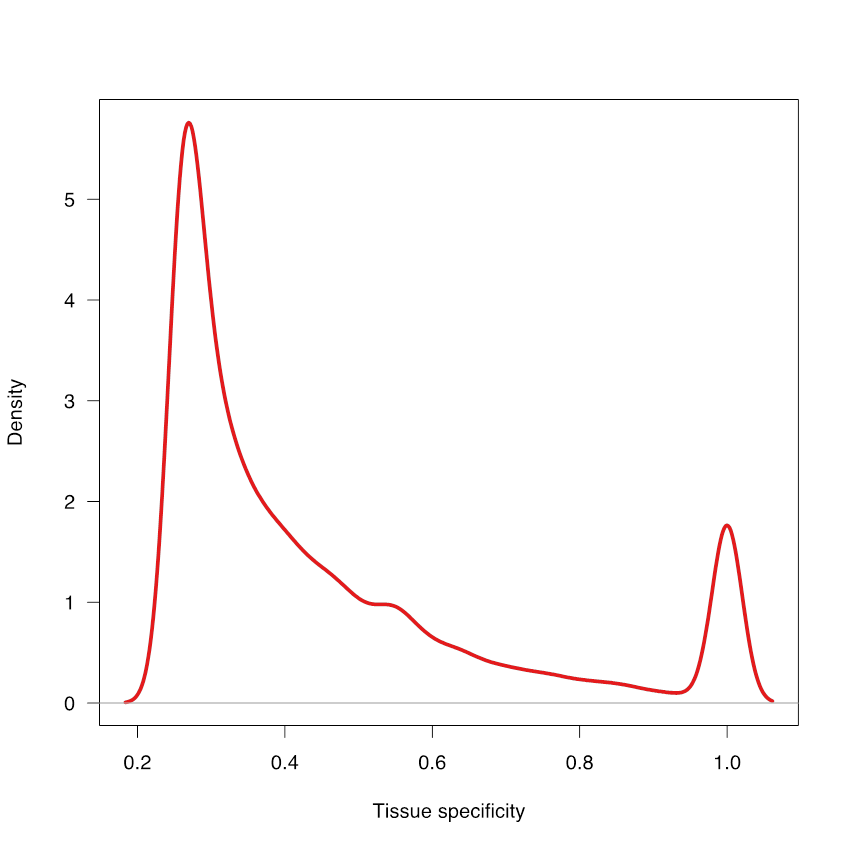


**Tissue specificity of human protein-coding genes.** The tissue-specificity scores of protein-coding genes using the human body map data (http://www.ensembl.info/blog/2011/05/24/human-bodymap-2-0-data-from-illumina/) were shown.

Supplementary Figure S6. Comparison of the tissue-specificity at a fixed expression level.

**Comparison of the tissue-specificity at a fixed expression level** (-2 < log(RPKM_max_) < 2). The tissue-specificity scores of novel lncRNAs compared with those of annotated lncRNAs and protein-coding genes.

# Supplementary Figure S7. RT-PCR validation of *S. scrofa* lncRNA expression levels.

**a**


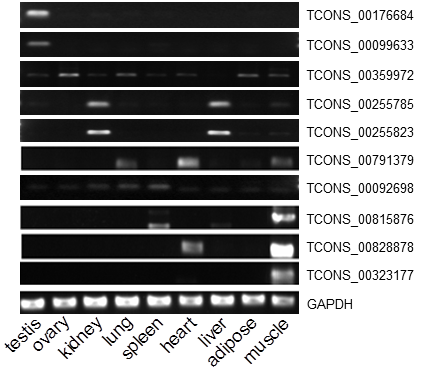


**b**


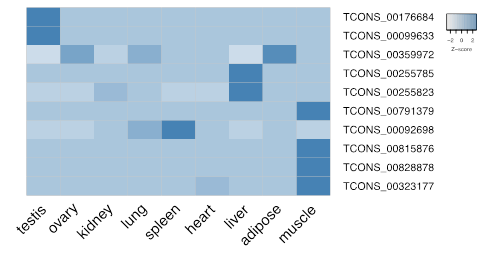


**RT-PCR validation of *S. scrofa* lncRNA expression levels.** (**a**) Validation of the expression of l0 lncRNA by RT-PCR in 9 pig tissues. (**b**) Heatmap of the relative expression (Z-score) of the validated lncRNA based on the RNA-seq data The expression level was determined by the RPKM value in each tissue.

**Supplementary Figure S8. Conservation analysis of lncRNAs, mRNAs, and introns in protein-coding genes in *Sus scrofa*.**


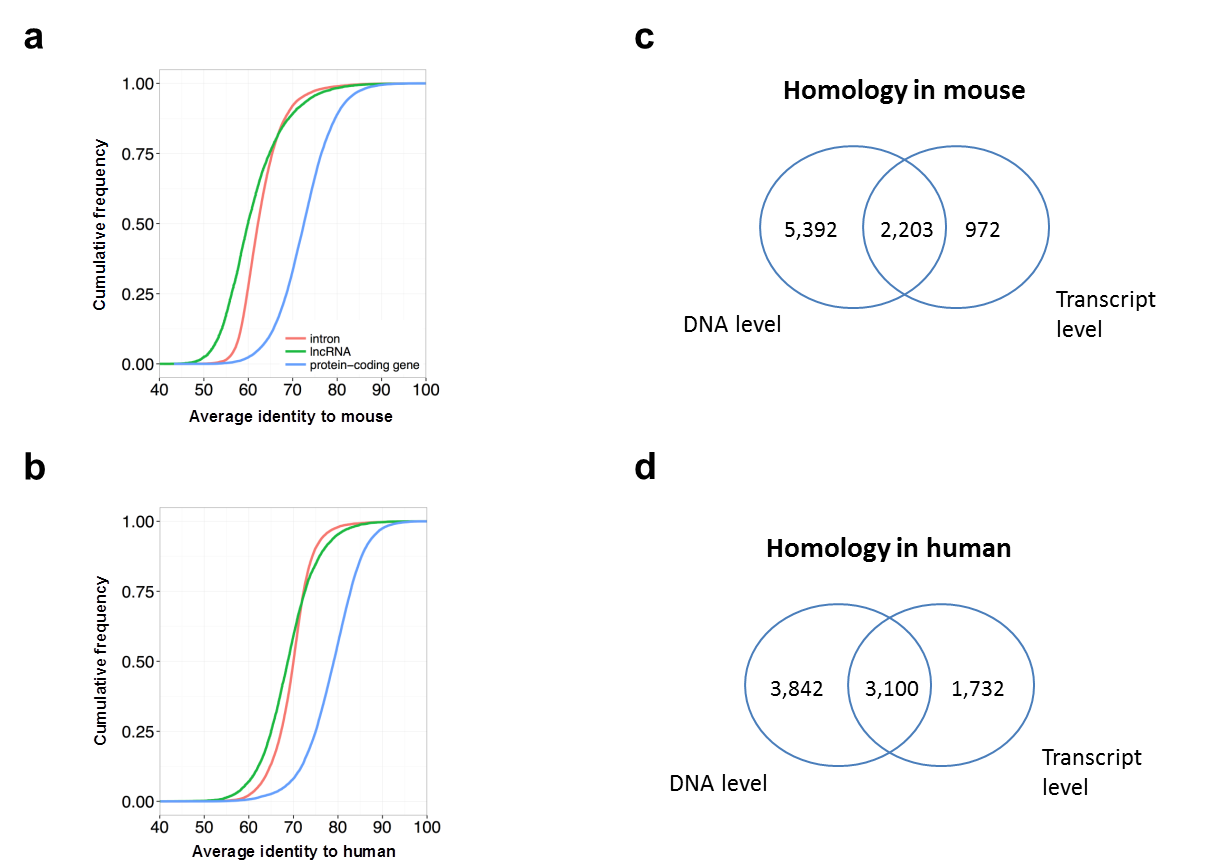


**Conservation analysis of lncRNAs, mRNAs and introns in protein-coding genes in *Sus scrofa***. (**a**) The conservation levels measured by the average identity to mouse of the three types of sequences in *Sus scrofa* are compared. (**b**) The conservation levels measured by the average identity to human of the three types of sequences in *Sus scrofa* are compared. (**c**) The comparison of conserved lncRNAs in mouse based on DNA level (genome alignment) and transcript level (BLAST to transcripts). (**d**) The comparison of conserved lncRNAs in human base on DNA level (genome alignment) and transcript level (BLAST to transcripts).

# Supplementary Figure S9. Temporal expression patterns of 6 selected lncRNAs during skeletal muscle development.

**Temporal expression patterns of 6 selected lncRNAs during skeletal muscle development.** For each lncRNA, the upper panel shows the RPKM values according to the RNA-seq data across the 3 developmental stages of skeletal muscle (orange line), and the bottom panel shows the relative quantitation values (RQ) according to the RT-qPCR experiments across the 3 developmental stages of skeletal muscle (blue line).

# Supplementary Figure S10. Differential splicing analysis of protein-coding genes in *Sus scrofa.*

**Differential splicing analysis of protein-coding genes in *Sus scrofa*.** (**a**) Statistics of the five types of alternative splicing events in protein-coding genes across 3 developmental stages of skeletal muscle, including mutually exclusive exons (MXE), alternative 5' splice sites (A5SS), alternative 3' splice sites (A3SS), retained introns, (RIs)and skipped exon (SEs). (**b**) GO enrichment of the protein-coding genes with differential alternative splicing events during skeletal muscle development. As the GO annotation for the genes in *Sus scrofa* is relatively limited, we converted the gene IDs into their human homologs based on the TreeFam database^1^, and performed GO enrichment analysis based on human annotation using the DAVID web server^2^.

# Supplementary Figure S11. Sanger sequencing results of a differentially spliced lncRNA, TCONS_00558282.


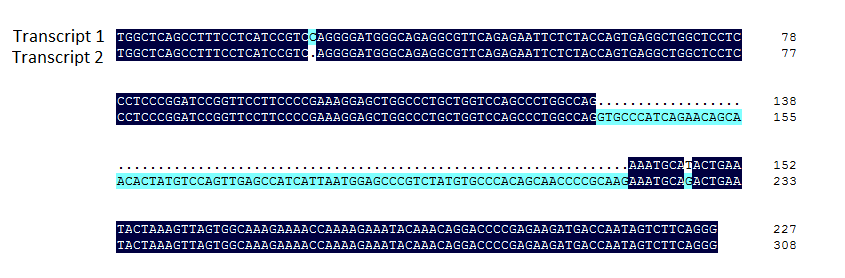


**Sanger sequencing results of a differentially spliced lncRNA, TCONS_00558282**. Transcript 1 is the shorter transcript with the third exon skipped (the lower band) in Figure 4e). Transcript 2 is the longer transcript with the third exon included (the upper band in Figure 4e).

# Supplementary Figure S12. Supporting information of the weighted co-expression network.


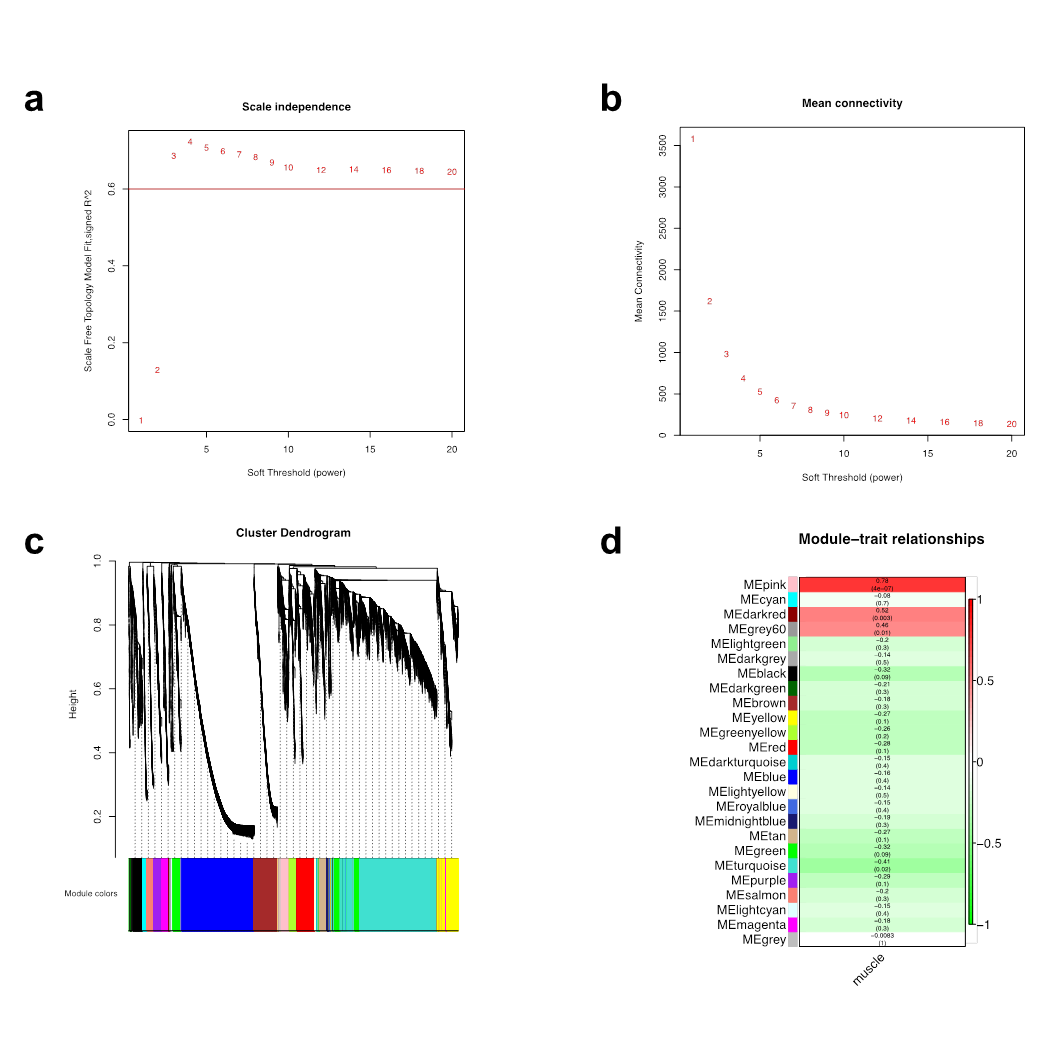


**Supporting information of the weighted co-expression network. (a)** We used a power of 6 and showed the scale free fit index as a function of power**. (b)** We showed the mean connectivity in the network at a function of power. **(c)** Clustering dendrogram of genes, with dissimilarity based on topological overlap, together with assigned module colors**. (d)** Each row corresponds to a module eigengene, column to the muscle trait. Each cell contains the corresponding correlation and p-value. The table is color-coded by correlation according to the color legend.

# Supplementary Figure S13. Comparison among newly predicted lncRNAs and previous studies.

**Comparison among newly predicted lncRNAs and previous studies.** The overlaps among our set, Zhao’s set^3^ and Zhou’s set^4^ were shown.

**Supplementary References**

1. Li, H. *et al.* TreeFam: a curated database of phylogenetic trees of animal gene families. *Nucleic Acids Res* **34**, D572-80 (2006).

2. Huang, D.W. *et al.* DAVID Bioinformatics Resources: expanded annotation database and novel algorithms to better extract biology from large gene lists. *Nucleic Acids Res* **35**, W169-75 (2007).

3. Zhao, W. *et al.* Systematic identification and characterization of long intergenic non-coding RNAs in fetal porcine skeletal muscle development. *Sci Rep* **5**, 8957 (2015).

4. Zhou, Z.Y. *et al.* Genome-wide identification of long intergenic noncoding RNA genes and their potential association with domestication in pigs. *Genome biology and evolution* **6**, 1387-92 (2014).
